# A Review of Biomedical Centrifugal Microfluidic Platforms

**DOI:** 10.3390/mi7020026

**Published:** 2016-02-06

**Authors:** Minghui Tang, Guanghui Wang, Siu-Kai Kong, Ho-Pui Ho

**Affiliations:** 1 Department of Electronic Engineering, The Chinese University of Hong Kong, Shatin, Hong Kong, China; mhtang@ee.cuhk.edu.hk; 2 Institute of Optical Communication Engineering, Nanjing University, Jiangsu 210009, China; wangguanghui@nju.edu.cn; 3 School of Life Sciences, The Chinese University of Hong Kong, Shatin, Hong Kong, China; skkong@cuhk.edu.hk

**Keywords:** centrifugal microfluidics, lab-on-a-disc (LOAD), biomedical, point-of-care diagnostics, cells, blood, nucleic acid, immunoassays

## Abstract

Centrifugal microfluidic or lab-on-a-disc platforms have many advantages over other microfluidic systems. These advantages include a minimal amount of instrumentation, the efficient removal of any disturbing bubbles or residual volumes, and inherently available density-based sample transportation and separation. Centrifugal microfluidic devices applied to biomedical analysis and point-of-care diagnostics have been extensively promoted recently. This paper presents an up-to-date overview of these devices. The development of biomedical centrifugal microfluidic platforms essentially covers two categories: (i) unit operations that perform specific functionalities, and (ii) systems that aim to address certain biomedical applications. With the aim to provide a comprehensive representation of current development in this field, this review summarizes progress in both categories. The advanced unit operations implemented for biological processing include mixing, valving, switching, metering and sequential loading. Depending on the type of sample to be used in the system, biomedical applications are classified into four groups: nucleic acid analysis, blood analysis, immunoassays, and other biomedical applications. Our overview of advanced unit operations also includes the basic concepts and mechanisms involved in centrifugal microfluidics, while on the other hand an outline on reported applications clarifies how an assembly of unit operations enables efficient implementation of various types of complex assays. Lastly, challenges and potential for future development of biomedical centrifugal microfluidic devices are discussed.

## 1. Introduction 

In past few decades, the lab-on-a-chip system, which aims at integrating laboratory work into a chip platform, has attracted a great deal of attention. The miniaturization of the lab-on-a-chip system allows bioassays to be implemented with decreased reagent use, a decreased total processing time, and increased abilities for parallel processing. In addition, the automation of lab-on-a-chip devices requires no skilled worker, and the absence of error-prone manual laboratory protocols makes the result of these assays consistent. Table 1 compares the different fluidics manipulation strategies in microfluidics. According to the source of the manipulating forces, these manipulation strategies can also be categorized as active and passive types. Active manipulation strategies, such as centrifugal microfluidics, electrokinetic microfluidics, magnetic microfluidics, optofluidics and surface acoustic wave microfluidics, rely on external force fields, whereas passive technologies depend entirely on the channel geometry or intrinsic hydrodynamic forces, such as capillary-driven test strips and inertial microfluidics.

As one branch of the lab-on-a-chip system, centrifugal microfluidic or lab-on-a-disc (LOAD) platforms exploit the centrifugal field to manipulate microfluidics. As the inherent centrifugal force exists everywhere on the disc and the direction is always radially outward, it acts just like a “gravity field”. This presents many advantages. For fluid transportation, centrifugal pumping requires only a compact motor and no external instrumentations. The fluid transportation in centrifugal microfluidic devices is also highly efficient and leaves no disturbing bubble or residual volume. In addition, density-based sample separation is inherently available. These advantages have not only contributed to an increase in centrifugal microfluidic research activity, but also attracted many companies to develop products based on centrifugal microfluidics. “Panasonic, Roche, Samsung, 3M, and Abaxis already have centrifugal microfluidic-based products on the market” [1].

Current reviews on the LOAD platforms are mainly focused on a specific topic in centrifugal microfluidics, for example handling and analysis of cell or bioparticles [2,3], molecular diagnostics [4], optical detection strategies [5] and detection methods [6]. While these reviews provide detail descriptions of specific developments in this field, a review on the overall development of LOAD may offer readers a concise summary of current development in centrifugal microfluidics. This is especially important for researchers who wish to extract information of the current development of the whole field. Until now, two comprehensive overviews on the topic of centrifugal microfluidic platforms have been published by Gorkin *et al.* [7] and Strohmeier *et al.* [1]. Despite that the second one was published at a time as recent as early 2015, a number of new biomedical centrifugal microfluidic devices and systems have been published since then. The new items include novel valving platforms (especially membrane deformation based valves and dissolvable films based valves), thermocycler based PCR platforms, CD4+ cells isolation and counting platforms, and isothermal amplification based platforms (especially loop mediated isothermal amplification and recombinase polymerase amplification based platforms), and centrifugal microfluidic techniques have also been used by researchers for detecting the dengue virus. This paper provides a comprehensive and up-to-date account on recent advances in biomedical centrifugal microfluidic platforms. Based on our analysis, we have come up with a list of challenges and outlooks that might inspire potential interests.

**Figure 1:**
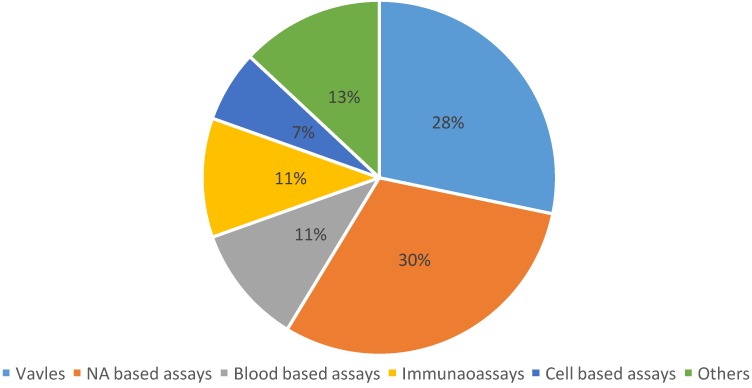
Distribution of topics covered by papers (total of 46) published within the theme of lab-on-a-disc in 2015.

Figure 1 summarizes the distribution of publications among current topics in biomedical LOAD platforms. It can be seen that NA (nucleic acid) based assays and valving systems are currently the most important topics. This signifies that researchers place equal emphasis on applications and device development. As the database suggests, exploration efforts are roughly classified into two categories: (i) unit operations that perform specific functionalities, and (ii) systems that aim to address certain biomedical applications. First, our overview on unit operations explains the basic concepts and mechanisms involved in centrifugal microfluidics. This is followed by an outline of biomedical applications that clarifies how an assembly of unit operations may enable efficient implementation of various complex assays. We have systematically classified biomedical applications based on the sample being used in the platform. This will help readers appreciate the rationale behind the choice of certain device strategies.

In centrifugal microfluidics, improvement of the complex bioassay devices depends largely on the development of several unit operations, including mixing, valving, flow switching, metering, and sequential loading. Effective sample mixing methods facilitate the physical or chemical reaction and decrease the time of the assay. Microvalves and flow switches make the fluid flow on the chip controllable and promise more possibilities and functionalities for the platform. Reagent volume metering makes the assays more accurate and repeatable. However, sequential loading of reagents is required in many biomedical applications such as immunoassays and DNA extraction. In this review, we introduce the development of these unit operations in detail. As centrifugal microfluidics primarily aims to address biomedical applications, reported efforts on nucleic acid analysis, blood analysis, immunoassays, and other biomedical tests are also covered. Finally, we present a conclusion to highlight recent research as well as outlook for future improvement.

**Table 1:** Comparison of different fluidic manipulation strategies in microfluidics in recent years.

Fluidic Manipulation Strategies	Principles	Applications	Strengths	Challenges
Capillary-driven test strips	Passive liquid transport via capillary forces within the capillaries of a fleece or a microstructured layer.	Diabetes testing; pregnancy testing; PH measurement; immunoassays; point-of-care diagnostics.	Cheap, small, and disposable; does not need any energy supply.	Precision of the assay is limited; stability of coating and or surface activations cannot be guaranteed after longtime storing [8].
Centrifugal microfluidics	Using centrifugal forces to process samples and reagents.	Nucleic acid analysis; blood analysis; immunoassays; point-of-care diagnostics.	Minimal amount of instrumentations; efficient removal of any disturbing bubbles or residual volumes; parallelization is available.	Large-scale integration is difficult; contact-free interface is not applicable during the assay [1,7,8].
Electrokinetic platforms	Based on surface forces and gain impact within the micro-dimensions due to the increased surface-to-volume ratio.	DNA and protein quantification; analytical chemistry field.	Pulse-free pumping without any moving part; enables the automation and parallelization of tests.	Need for high performance detection technologies and high voltages [8].
Droplet based microfluidic platforms	Use of single droplets as reaction confinements for biological assays or chemical reactions.	Fabricate special materials; screen and analyze biomedical or chemical reaction products.	Decreasing reagent and sample consumption; many same-size droplets means test can be repeated.	Device fabrication is difficult; manipulation droplet flexibility is tough; better understanding of the dynamics in droplets is needed [9].
Digital microfluidics	Use electrostatic forces to manipulate discrete droplets.	Sample preparation or extraction; blood analysis; DNA analysis; cell analysis; immunoassays.	Enables precise, real time and high flexible control without need for pumps, valves.	Fail at high temperatures and pressures; manipulating concentrated samples is difficult; dielectric breakdown with high voltage usage [10,11,12,13].
Surface acoustic wave microfluidics	Use of 10–1,000 MHz acoustic waves to manipulation microscale fluid.	Biomolecular and cellular manipulation and detection, drug delivery, biomaterials synthesis, and point-of-care diagnostics.	High biocompatibility, fast fluid actuation, versatility, compact, and inexpensive; delivers a complete microfluidics solution at the microscale.	Physics of SAW microfluidics are not understood completely; unsolved problems in practical applications, e.g., deformation of the fluid interface [14,15].
Inertial microfluidics	Use inertial migration and secondary flow caused by the inertia of the fluid to manipulate particles.	Cellular sample processing; blood plasma extraction; particles sorting.	Enables high-throughput, simple, precise and low cost manipulation.	Quantitative design rules are still lacking for channels; separation resolution and processing speed should be improved [16,17,18,19,20].

## 2. Physics in Centrifugal Microfluidics 

To better understand the principle of the manipulations in centrifugal microfluidics, we introduce the basic physical background for this topic as follows.

Centrifugal force, which acts radially outward on the disc, provides the centripetal acceleration during the rotation process. The centrifugal force is:

(1) $$f_{ce}=m\omega^{2}r$$

where m is the mass of sample in the channel, r is the position on the disc, and ω is the angular rotational frequency. The pressure at the far end of a radial column of incompressible fluid (density ρ) extending from radius $r_{1}$ to $r_{2}$ is:

(2) $$p_{ce}=\int_{r_{1}}^{r_{2}}{\rho \omega }^{2}rdr=\frac{1}{2}{\rho \omega }^{2}\left(r_{2}^{2}-r_{1}^{2}\right).$$

In centrifugal microfluidics, there are two other forces based on rotation: the Coriolis force ($f_{co}$) and Euler force ($f_{e}$). The Coriolis force is velocity dependent and given by:

(3) $$f_{co}=-2m\omega \times v$$

The Euler force is given by:

(4) $$f_{e}=-m\frac{d\omega }{dt}\times r$$

It is proportional to the angular acceleration and exists only when the rotation acceleration is not zero.

In addition to these three forces, viscous, capillary, and fluidic inertia force are not based on rotation but play important roles in centrifugal microfluidics. The pressure corresponding to these three forces $\Delta p_{vi}$, $\Delta p_{ca}$, and $\Delta p_{in}$ are given by [1]:

(5) $$\Delta p_{vi}=-R_{hyd}q$$

(6) $$\Delta p_{ca}=\sigma \kappa$$

(7) $$\Delta p_{in}=-\rho \ell a$$

$R_{hyd}$ is the hydraulic resistance and is proportional to the dynamic viscosity, q is the volumetric flow rate, σ denotes the surface tension of a processed liquid, κ denotes the curvature of its meniscus, ℓ is the length of a fluidic channel filled with the liquid, and a is the acceleration of the liquid.

On the disc, the laminar flow fluids often experience repeated transporting, mixing, and recombination in the channels or chambers. We often want to know the velocity vector of the fluid flow in addition to the concentration distribution in the channels or chambers. The velocity vector and concentration distribution can be calculated using Equations (8) and (9), respectively [21].

(8) $$\rho \left(\frac{\partial \vec{u}}{\partial t}+\vec{u}\cdot \nabla \vec{u}\right)=\mu \nabla^{2}\vec{u}-\nabla p+\vec{F}$$

(9) $$\frac{\partial C}{\partial t}+\nabla \cdot \left(\vec{u}C\right)=D\nabla^{2}C+R_{g}$$

ρ is the density of the fluid, $\vec{u}$ denotes the velocity vector of the fluid, μ is the dynamic viscosity of the liquid, ∇p is the pressure gradient, $\vec{F}$ is the external force field applied to the fluid (e.g., centrifugal force), $\nabla \cdot \left(\vec{u}C\right)$denotes convective mass flux, $D\nabla^{2}C$ denotes diffusive mass flux (*D* is the diffusion coefficient), and $R_{g}$ is the net rate of the species generation.

## 3. Advanced Unit Operations 

The unit operations refer to the basic building blocks used in centrifugal microfluidic complex applications. An introduction of the advanced unit operations should give readers deeper insight into their mechanisms and pave the way for a better understanding of the later complex applications. The advanced unit operations include sample mixing, valving, switching, metering, and sequential loading. The merit of these unit operations makes the centrifugal microfluidic platform more powerful.

### 3.1. Mixing

Mixing is performed to reach a sufficient homogeneity and increase the contact surface area of reaction reagents, thereby decreasing the reaction time. On the centrifugal microfluidic platform, because the scale of the channel is very small and mixing efficiency depends largely on the contact area, purely diffusive mixing in not enough for applications [1,22,23]. Conventional diffusive mixing in chambers that are several millimeters wide typically takes place on a timescale of minutes [24], and effective mixing methods usually reach homogeneity within several seconds [25]. Therefore, methods for increasing the efficiency of mixing on the centrifugal microfluidic platform have attracted increasing attention. We classify these methods into two groups: those based on intrinsic forces and those based on external perturbations.

#### 3.1.1. Mixing Based on Intrinsic Forces

Intrinsic forces including centrifugal force, the Coriolis force, and the Euler force are induced merely by the presence of centrifugation. Effective mixing is often accomplished using the Euler and Coriolis forces.

Grumann *et al.* demonstrated a mixing method based on the Euler force. Here, Euler force was induced by changing the rotation speed of the disc periodically. As a result, a shear-driven advective current arose within the chamber. This strategy decreased the mixing time from about 7 min for mere diffusion to less than 5 s [22].

Haeberle *et al.* demonstrated a mixing method based on the Coriolis force. Two liquids were initially injected into two separated channels and then merged in a Y-shaped channel. The Coriolis force acted perpendicular to the flow direction and then caused transversal convection in the channel, which accelerated the mixing process [26]. Kuo *et al.* combined channel geometry and the Coriolis force to make a more efficient mixing method. Numerical simulations were performed to investigate the mixing performance of three CD microfluidic mixers with square-wave, curved, and zigzag microchannels with the help of the Coriolis force, and the square-wave microchannel was found to exhibit the best mixing performance [27].

#### 3.1.2. Mixing Based on External Perturbations

In addition to the mixing methods based on intrinsic forces during centrifugation, some mixing methods have been based on external perturbations.

**Figure 2:**
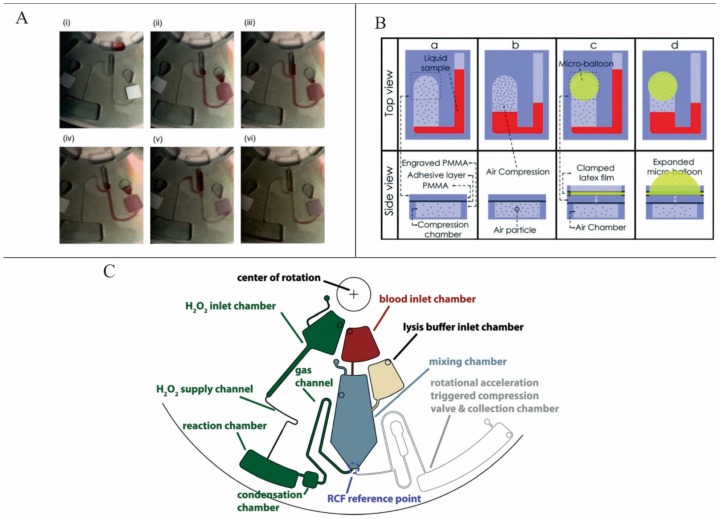
(**A**) Bidirectional flow caused by pneumatic energy and centrifugal force. Figure reprinted with permission from [28]. (**B**) Conventional pneumatic pumping and latex micro-balloon pumping. Figure reprinted with permission from [29]. (**C**) Buoyancy driven bubble mixing. Figure reprinted with permission from [30].

Noroozi *et al.* demonstrated a micro-mixing method based on bidirectional flow. In this system, centrifugal acceleration leading a liquid element to extrude air first generated and then stored pneumatic energy. The pneumatic energy was released by a reduction of the centrifugal speed and then the direction of the liquid flow was reversed (Figure 2A). In this way, the bidirectional flow was achieved. This system decreased the processing time and reagent consumption by one order of magnitude [28]. To make the pneumatic energy storage more effective, Aeinehvand *et al.* presented a system that used a latex micro-balloon to pump liquid against the centrifugal force (Figure 2B). It proved that the micro-balloon could operate at lower rotational speeds and could pump a larger volume of liquid toward the center of the disc [29]. However, these two methods depended too much on the change of the centrifugation speed. Kong *et al.* demonstrated a technique that allowed mixing without the need to alter the rotational frequency or direction. Here, an external air stream was also used to agitate liquids and then facilitate the mixing process [25]. Grumann *et al.* presented a method using magnetic beads to accelerate the mixing process. A deflection of the magnetic beads was induced by “a set of permanent magnets equidistantly aligned at spatially fixed positions in the lab-frame” [22]. Burger *et al.* also presented a bubble mixing LOAD platform (Figure 2C). Here, air (oxygen) was generated by the decomposition of hydrogen peroxide and mixing was improved by the rupture of the bubbles which caused a strong buoyancy in centrifugal field [30].

### 3.2. Valving 

Valving is one of the most important basic functions in centrifugal microfluidics. It is used to control the fluid movement in the following channel networks. In complex application devices, valves are often used to realize the sequential loading of pre-stored reagents. We group the valves into capillary, hydrophobic, siphoning, centrifugal-pneumatic, and active valves.

#### 3.2.1. Capillary and Hydrophobic Valves

The capillary valve is based on the competition between two forces: centrifugal force and the capillary force. When the centrifugal pumping pressure is smaller than the capillary barrier, the fluid will not pass the capillary valve. Therefore, an increase or decrease in the rotation speed can switch the capillary valves in the practical applications on or off. These capillary valves are used in a variety of biochemical applications [8,31,32,33,34]. Many researchers have also investigated the influence of the microchannel dimensions, surface tension, and contact angle of the liquid in capillary valving [35,36,37,38].

Al-Faqheri *et al.* presented pneumatic assisted capillary centrifugal microfluidic valving system. At low rotation speed, as the capillary pressure, centrifugal pressure, and pneumatic pressure were balanced, liquid was stopped in the valve. At high rotation speed, the increased the centrifugal pressure drove the liquid into the subsequent chamber [39].

Local hydrophobic surface coatings are sometimes applied or a channel is suddenly narrowed to increase the capillary pressure. These valves are often called “hydrophobic valves” and have been the subject of much research [40,41]. For some other reagents, local hydrophilic surface coating is sometimes applied or a channel is suddenly widened to increase the capillary force. These valves are correspondingly known as “hydrophilic valves”.

#### 3.2.2. Siphoning Valves

As siphoning relies on the priming of liquid into a siphon channel due to the capillary action, the siphon channel surface must be hydrophilic [7]. The system in a siphoning valve spins at a high speed to prevent capillary priming. When the rotation speed decreases, the capillary pressure exceeds the pressure caused by centrifugal force and the fluid passes the crest point of the siphon. Consequently, the siphon valve is switched on.

**Figure 3:**
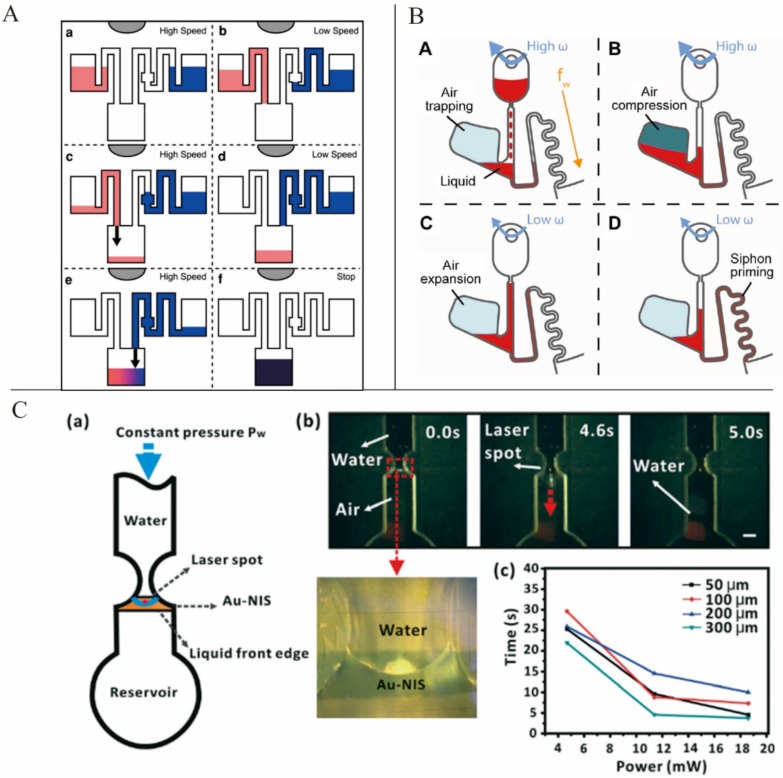
(**A**) Sequential loading of fluids by serial siphon valves. Figure reprinted with permission from [42]. (**B**) Centrifugo-pneumatic siphoning. Figure reprinted with permission from [44]. (**C**) Optofluidic valving. Figure reprinted with permission from [48].

Siegrist *et al.* introduced a novel serial siphon valve that relied on multiple inline siphons to provide for a sequential loading of fluids (Figure 3A). This design allowed the fluids to be subsequently distributed to a designated area through multiple acceleration and deceleration operations [42]. Burger *et al.* presented an overflow siphon valve where liquid filled the chamber and was held back by the outlet siphon until the crest point of the siphon was reached [43]. Godino *et al.* presented a centrifugo-pneumatic siphon system. Siphon priming was achieved via the release of pneumatic energy from an enclosed and compressed air bubble [44] (Figure 3B). Keller *et al.* reported a centrifugo-thermopneumatic (CTP) siphon valve. The cartridge was used in an off-the-shelf centrifugal thermocycler to control the global temperature. When the temperature increased, gas expanded and drove the liquid to pass the crest point of the siphon. In this paper, CTP two-stage aliquoting was also demonstrated [45].

Zehnle *et al.* presented three pneumatic siphon valving platforms: rotational frequency-triggered vacuum valve (RFT-VV), rotational acceleration-triggered vacuum valve (RAT-VV), and rotational acceleration-triggered compression valve (RAT-CV). In RFT-VV, the increased centrifugal force drove the liquid level in the siphon to reach the siphon crest. In RAT-VV, because of Euler force which was induced by fast acceleration, the siphon was primed. While, in RAT-CV, the siphon valve was open by the combination of pneumatic force and Euler force [46].

#### 3.2.3. Active Valves

Active valves are valves that can be switched on or off by external means during an experiment. The most commonly used active valves are paraffin wax valves. In this kind of valving system, the channel is barred by paraffin wax, which can be melted easily. Stationary infrared sources are often used to heat the wax and thus switch on these valves during experiments. Park *et al.* presented a novel paraffin wax valve in which iron oxide nanoparticles were mixed into the wax, allowing for valve actuation via low-power lasers (1.5 W) and a response time of only 0.5 s [47]. In addition to the paraffin wax valve, laser-beam-activated valves have been introduced into centrifugal microfluidic platforms. Chen *et al.* demonstrated a type of optofluidic valving that used laser beams to illuminate the water meniscus and forced the water to burst into the subsequent channel [48] (Figure 3C).

Swayne *et al.* presented a hydrocarbon gel-based valve. Liquid flow was restricted by the hydrocarbon gel plug. A modified pneumatic system was used to disperse the plug and rendered opening of the valve [49]. Cai *et al.* fabricated a membrane deformation based valving platform. At low spinning frequency, spring plungers were compressed against the bottom membrane of the valve and the valve was closed. When the spinning frequency increased, flyball governor drove the spring plungers away and got the valve open [50,51]. The same group also reported a similar magnetically actuated membrane deformation based valving system. Here, the valve was first sandwiched between two permanent magnets. At low spinning frequency, because of the magnetic force, the top membrane of the valve was compressed and the valve was closed. At high spinning frequency, the bottom-side magnet was driven away by fly ball governor and the valve was open [52]. Al-Faqheri *et al.* also reported a similar valving system based on membrane deformation, with the exception that the spring plunger or permanent magnet was replaced by air pressure to compress the membrane [53].

Aeinehvand *et al.* presented a reversible thermo-pneumatic valves (RTPV). This RTPV consisted of an air chamber and a liquid transition chamber. The air chamber was enclosed by a latex membrane, and when the trapped air volume was heated, this membrane expanded into the liquid transition chamber and sealed the inlet. With this operation, trapped air volume was heated or cooled to make RTPV closed or open, respectively [54].

**Figure 4:**
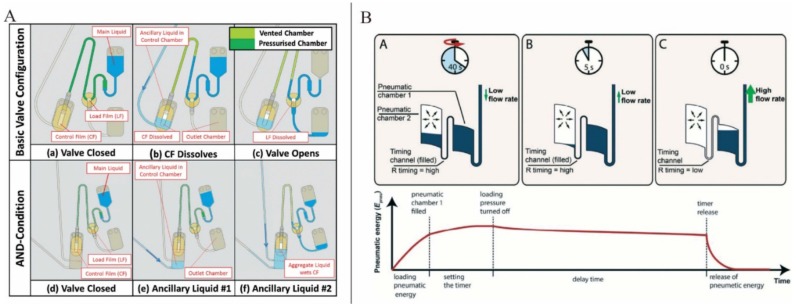
(**A**) Basic event triggered valve and AND-condition valve. Figure reprinted with permission from [56]. (**B**) Microfluidic timer. Figure reprinted with permission from [58].

Dissolvable films (DFs) sacrificial valving systems have attracted much attention. DFs will breakdown when a liquid is introduced to their surface, eventually allowing liquid to pass through the valve. This operation principle makes DFs valving independent of the rotation speed and manufacturing tolerances. Gorkin *et al.* presented an advanced DFs valving system in which a pneumatic chamber was used in combination with DFs. Here, when the rotation speed reached a critical value, the liquid could break the barrier of the air in the pneumatic chamber and wetted the DF membrane [55]. Kinahan *et al.* presented a centrifugo-pneumatic DF valving system which consisted of a pneumatic chamber sealed by two DFs: the load film (LF) and the control film (CF) (Figure 4A). The pneumatic chamber closed by CF membrane was used to prevent the restrained liquid from contacting and dissolving the LF at typical spin speeds. However, when an ancillary liquid was introduced, the CF membrane would be dissolved and the restrained liquid would enter the pneumatic chamber, wet and dissolve the LF and thus would open the valve. Furthermore, based on this centrifugo-pneumatic DF valving scheme, AND-condition actuation was also suggested. In AND-condition actuation, CF membrane could only be wetted by the presence of the aggregate volume of at least two ancillary liquids. Lastly, event-triggered valving system in which the arrival of liquid from the first valve at defined locations prompted the opening of the second valve was also proposed [56]. However, in this event-triggered valving system, the interval between subsequent valve actuations was not suffice for some laboratory unit operations such as extended mixing or biological incubation steps. To solve this problem, this same group suggested another paper imbibition event-triggered system. Here, ubiquitous paper strip was used to transport liquid to the CF and because of the material-specific speed of imbibition, the interval between valve openings was defined by the spacing between CFs [57]. Schwemmer *et al.* also presented a timed valving and pumping LOAD platform (Figure 4B). The timer was based on the time delay in releasing compressed air [58].

### 3.3. Flow Switching

In many complex centrifugal microfluidic biomedical applications, reagents must often be directed into one of several channels or chambers. On this occasion, flow-switching technology is essential.

The most common flow-switching method is based on the Coriolis force [59,60,61]. The switching of the fluids often takes place in Y-shape channels and depends on the rotation direction and the corresponding Coriolis force. However, there are also active flow-switching methods.

Zehnle *et al.* presented a rotational acceleration-triggered compression switching system. Here, the switching was based on the magnitude of Euler force, not the direction of Euler force [46]. Kong *et al.* presented an active flow-switching method using a regulated stream of compressed gas. This pneumatic flow-switching method allows for flow control at a T-shaped junction between one inlet channel and two outlet channels [62]. Chen *et al.* demonstrated an optofluidic switching method, according to which the fluidic channel was blocked by two valves along the “Y” junction and then one chosen valve was opened by a 20-mW 785-nm laser beam [48]. Although these active switching methods can be carried out successfully, they often require external devices, which makes the assay complex and difficult to implement.

### 3.4. Metering and Sequential Loading

**Figure 5:**
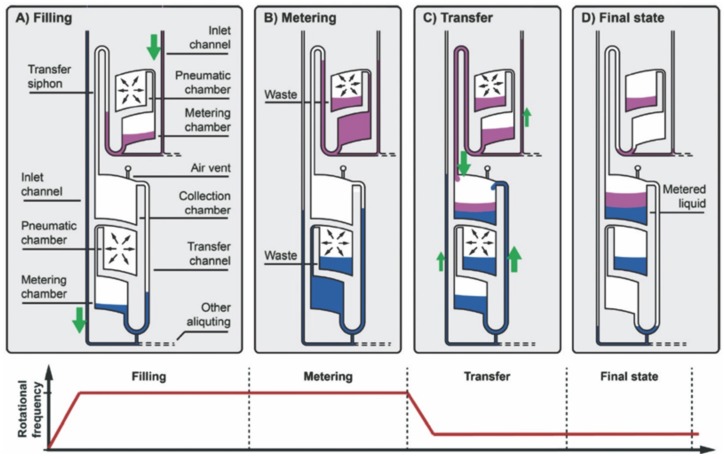
Principle of centrifugo-pneumatic metering. Figure reprinted with permission from [63].

The reagent volume often greatly influences the diagnostic result. Furthermore, the repeatability of the system and valving process depends largely on the reagent volume. Thus, liquid volume metering plays an important role in centrifugal microfluidics. Metering in centrifugal microfluidics is often achieved based on a structure in which an overflow waste chamber is connected to a metering chamber with a defined volume [1]. However, the metering accuracy of this structure often deteriorates due to the wicking effects at liquid interfaces caused by capillary forces. Steigert *et al.* introduced design principles to counteract this effect and achieved a metering of 300 nL with a 5% variability coefficient [63]. Schwemmer *et al.* presented a platform in which liquid metering, aliquoting of multiple liquids and subsequent pairwise combination were demonstrated with the help of centrifugal forces and pneumatic forces (Figure 5). Here, at a high rotation frequency, air in pneumatic chambers was compressed and liquid in the metering chamber was metered. When rotational frequency was quickly decreased, the compressed air pushed the metered liquids into the mixing chamber [64].

Sequential loading is also an essential function in centrifugal microfluidics, as the reagent-adding sequence is of great importance in many reactions or diagnosis, especially in nucleic acid extraction and immunoassay protocols. Sequential loading means that the fluids are driven into a designated chamber in a defined order. In Section 3.2.2, we present a sequential release of fluids based on multiple inline siphons (Figure 3A).

Park *et al.* presented a system in which different dimensional microfluidic channels (120, 40, and 20 μm) were used as passive capillary valves with different thresholds. Centrifugal force controlled the loading of the reagents in sequence. This sequential loading system was used for H1N1 viral RNA purification [65]. Jung *et al.* presented a similar system using capillary valving and a siphon channel simultaneously to realize the sequential loading [66].

Ukita *et al.* recently presented a water-clock-based autonomous sequential release of reagents in a steadily rotating centrifugal microfluidic device. The flow modes to the sample chambers were switched by supplying the air to the sample chambers [67]. However, in this method, transparent adhesive tape must be used to seal all of the chambers before the assay.

## 4. Current Biomedical Applications Based on Centrifugal Microfluidics Technology

### 4.1. Nucleic Acid Analysis

Conventional bench-top nucleic acid analysis is often time consuming and requires skilled workers and relatively expensive equipment. With the development of centrifugal microfluidics technology, all of these bench-top workflows can be integrated into one disc-like chip, decreasing the laboratory time and reagent and equipment costs to a large extent. This technology also makes the point-of-care based on nucleic acid analysis more accessible, as it requires minimal resources and no special laboratory training. The review in this section is presented based on two major steps in nucleic acid analysis: nucleic acid extraction and amplification.

#### 4.1.1. Nucleic Acid Extraction

The extraction of nucleic acid often involves two steps: lysis of eukaryotic or bacterial cells and nucleic acid purification.

Various forms of lysis systems are used and can be roughly classified into two main methods: chemical/biological and physical methods. For the chemical/biological methods, alkaline buffers [68] or enzymes are often used to decompose the membranes or cell walls to release DNA. As no external device is needed, chemical/biological methods are the simplest to implement. However, these methods often leave behind residual substances that inhibit subsequent processes. Microfluidic physical lysis methods including electrical, laser, and mechanical methods often require additional instrumentation. Like the commonly used physical method, mechanical lysis leaves behind little or no residual reagents and is faster and more efficient, especially for Gram-positive microbes, which have thick cell walls.

Kido *et al.* demonstrated a mechanical method that used the relative motion of ferromagnetic metal discs and grinding matrices in a liquid medium within individual chambers of the disc. In this system, an oscillating magnetic field that produced mechanical impaction and shear forces disrupted cells within the chamber. Glass beads were also integrated into each lysis chamber within the disc to make the lysis more effective. This system yielded clarified lysates from *E. coli* and *S. cerevisiae* using up to 70 μL of the substrate [69]. Kim *et al.* presented a beads beating lysis system in which the rotation direction was alternated to enhance the collisions and shearing between the beads and cells in the chamber [70]. Cho *et al.* introduced a rapid cell lysis system using laser irradiation on magnetic particles [71]. The instantaneous change in temperature induced by the laser facilitated the lysis of the cell.

Park *et al.* demonstrated a microsystem for automatic RNA purification. In this system, the virus sample, washing solution, and elution buffer were sequentially loaded into the sol–gel chamber and then viral RNA captured by the sol–gel solid phase was purified and eluted in 5 min. The RNA capture yield was measured as 80%, and the H1 and M genes were successfully amplified from the recovered purified H1N1 viral RNA by reverse-transcriptase PCR [65]. Jung *et al.* presented a similar sequential loading system to purify the influenza A H3N2 viral RNA. In that case, the sol–gel solid phase was replaced by a microbead-bed microchannel to capture the RNA. Overall, 81% of the RNAs were successfully captured and purified in 440 s [66]. Dimov *et al.* presented a solvent-selective routing technique for RNA purification. Here, the routing operation was realized based on the combination of hydrophobic membrane valve (HMV) and dissolvable film valve (DFV) [72].

**Figure 6:**
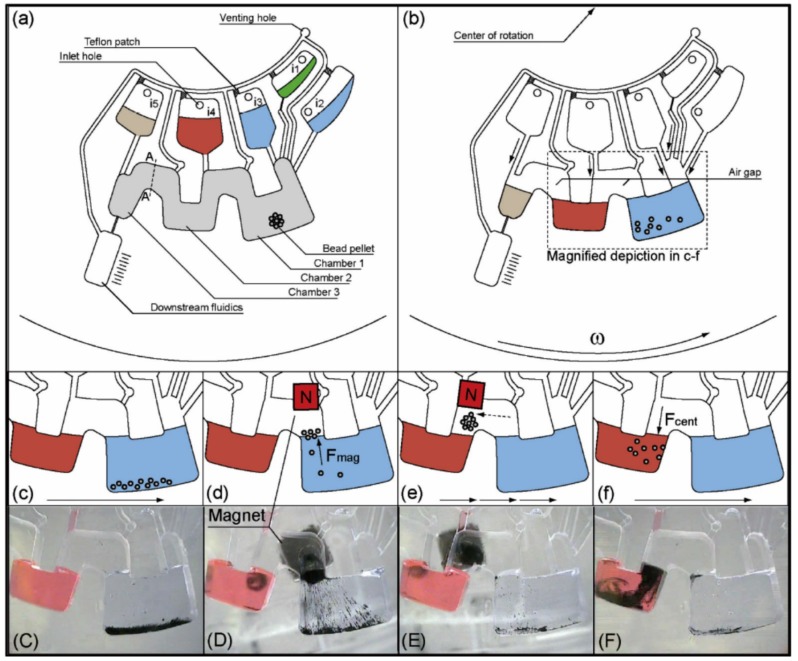
Gas-phase transition magnetophoresis system for DNA purification. Figure reprinted with permission from [73].

Strohmeier *et al.* presented a gas-phase transition magnetophoresis system for DNA purification from a dilution series of a *L. innocua* lysate and from a *lambda phage* DNA standard (Figure 6). In this system, a stack of stationary permanent magnets was used to help transport magnetic beads between three microfluidic chambers on a centrifugal microfluidic cartridge [73]. This method did not require any human interaction and could be adjusted for other applications easily. Later, the same group reported a platform capable of performing lysis and nucleic acid purification within the same cartridge [74].

#### 4.1.2. Polymerase Chain Reaction (PCR)

As a sensitive and specific method, nucleic acid-based detection offers several advantages over traditional microbiological detection for point-of-care clinical diagnostics or on-site detection of environmental, foodborne, and water-borne pathogens [75]. However, the limitation of the amount of nucleic acids in the target often impedes detection in the early stages. 

As a nucleic acid amplification method, polymerase chain reaction (PCR), which requires different temperatures that typically range between 55 and 95 °C, is widely accepted because monitoring it in real time allows for the highly sensitive quantification of DNA down to the single molecule level [1]. Through the PCR process, the target nucleic acid is amplified into quantities large enough for detection by fluorescence or electrochemical methods [76,77].

In recent years, the integration of PCR into centrifugal microfluidic cartridges has attracted much attention, as the enclosed microsystems decrease the risk of cross-contamination and the miniaturization decreases the volume of expensive amplification reagents and the thermal mass to ensure precise temperature control. Based on the thermocycling mechanism, the centrifugal microfluidic PCR system can be divided into two groups: chamber stationary PCR, where the target gene is amplified in a designate microchamber with a heater [78,79], and flow-through PCR, where the PCR occurs when the reagents circulate in different chambers at different temperatures [80].

**Figure 7:**
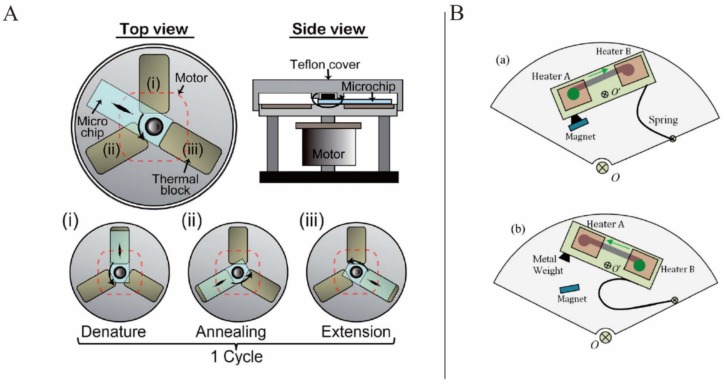
(**A**) PCR microchip sequentially rotated on the three thermal blocks to complete one PCR cycle. Figure reprinted with permission from [81]. (**B**) Speed actuated inertial mechanical structure: (**a**) low speed state, (**b**) high speed state. Figure reprinted with permission from [86].

Jung *et al.* presented a rotary PCR genetic analyzer to perform reverse-transcription PCR by combining the characteristics of the stationary PCR and flow-through PCR. Here, the PCR microchip was sequentially rotated on the three thermal blocks (the denaturation, annealing, and extension blocks) by a stepper motor to complete one cycle of PCR (Figure 7A). The fine tuning of the thermal block temperature and rapid rotation of the microchip between the thermal blocks enabled the target gene amplification in 25.5 min [81]. Stumpf *et al.* presented a sample-to-answer influenza A H3N2 virus detection platform in which the complete pathogen lysis, nucleic acid extraction, real-time reverse transcription polymerase chain reaction were demonstrated in less than 3.5 h [82].

Czilwik *et al.* presented a similar sample-to-answer molecular diagnostic platform for detection of bacterial pathogens [83]. Furutani *et al.* reported a LOAD platform to detect *S. enterica* in Food. Here, because of its low concentration, *S. enterica* was cultured on the same chip before PCR, which made the detection more sensitive and cheaper [84]. Keller *et al.* later presented a PCR platform based on a centrifugal real-time PCR thermocycler. Based on this platform, a PCR assay for identification of forensic animal family was demonstrated [85].

Wang *et al.* presented a solution for localized temperature cycling in PCR: an inertial mechanical system in which springs and magnets (Figure 7B) were used to achieve the bidirectional flow by changing the rotation speed. They also suggested adopting a lab-in-a-droplet bioassay strategy. With the help of this inertial mechanical structure, the bidirectional flow of the sample droplet between two temperature zones was demonstrated while the disc was spinning. The real-time heating in the designated area depended on a wireless power supply system that also made wireless programmable functionality possible [86]. Miao *et al.* also reported a double-shaft turntable LOAD platform to realize flow-through PCR and bidirectional flow [87].

In PCR, it is often the case that the system should be fully closed to avoid vapor loss and cross-contamination. Amasia *et al.* presented a centrifugal microfluidic system using ice valving to seal the thermocycling chamber and decrease fluid loss due to evaporation. The system was validated via amplification of a *B. anthracis* gene [77]. Furthermore, to solve the overpressure caused by high temperature evaporation in a closed thermocycling chamber during PCR, Czilwik *et al.* presented a method in which a microchannel was integrated as a vapor-diffusion barrier (VDB) to separate the liquid-filled PCR chamber from an auxiliary air chamber. In such configurations, as the process of vapor diffusion through the VDB was slowed down, the propagation of vapor from the PCR chamber into the auxiliary air chamber was limited. This system proved that at a temperature increase from 23 to 95 °C, overpressure decreased from more than 80 kPa to only 35 kPa with the use of the VDB [88].

#### 4.1.3. Isothermal NA Amplification Methods

Although PCR has the advantage of high sensitive and accuracy quantification, the thermal cycling makes the integration process difficult and expensive. To solve this problem, isothermal application methods such as loop mediated isothermal amplification (LAMP), recombinase polymerase amplification (RPA), rolling circle amplification (RCA), and helicase dependent amplification (HDA) are suggested.

**Figure 8:**
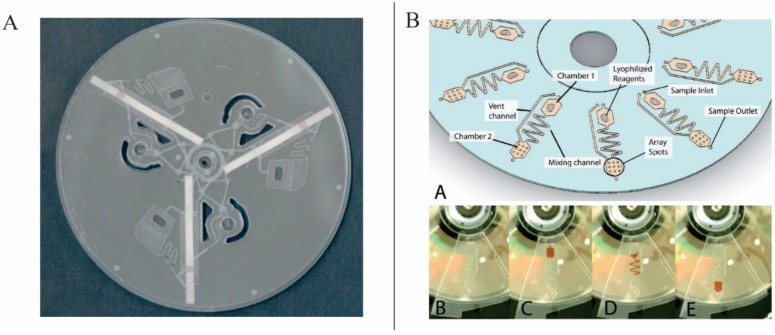
(**A**) RT-LAMP-ICS microdevice. Figure reprinted with permission from [91]. (**B**) Microfluidic DVDs for solid-phase RPA. Figure reprinted with permission from [96].

LAMP is a simple and sensitive NA amplification method that amplifies target genes at 60–65 °C within 1 h using polymerase enzyme and primer sets [89,90,91]. For the detection of the amplification products in LAMP, the usage of gel electrophoresis, turbidity or fluorescence signals, and colorimetric detection have been reported [6,89,90,91]. Among them, the rather cheap and simple colorimetric detection has been widely employed [89,91]. Oh *et al.* presented a foodborne pathogen identification platform which utilized LAMP for NA amplification and Eriochrome Black T (EBT) for colorimetric detection. Here, zigzag-shaped microchannels were used for sequential loading of the reagents [89]. Regarding the detection of virus, reverse transcriptase loop-mediated isothermal amplification (RT-LAMP) in which reverse transcriptase enzymes are added has also been developed. The same group demonstrated an integrated rotary genetic analysis microsystem in which RNA extraction from the influenza viral lysates, RT-LAMP, and real-time fluorescence detection were serially operated. Firstly, capillary calves and a siphon channel were used for sequential loading in RNA extraction. Then, the isolated RNA was driven into RT-LAMP reaction chamber by changing the rotation direction. In this system, using influenza A H1N1, H3N2, and H5N1 viral samples, the target gene (H1 and M genes) amplification was detected within 47 min [90]. This group also presented a similar influenza A virus identification platform in which immunochromatographic strip (ICS) was used for colorimetric detection instead of fluorescence detection to make the system more compact and portable (Figure 8A) [91].

Uddin *et al.* presented a LAMP detection system in which emitted florescence from labeled LAMP amplicons was detected by color sensor and the detection results were displayed on liquid crystal display (LCD) [92]. Santiago-Felipe also presented a real time LAMP monitor system. Here, LAMP was demonstrated on a standard audio-video disc and real time detection of the amplification products was done with the use of a disc readout laser [93].

RPA is another fast and simple DNA amplification technique which works at a constant temperature of ~39 °C [94]. However, quantitative measurements in RPA have still been a difficult problem so far. To solve this problem, Schuler *et al.* presented a first digital droplet recombinase polymerase amplification (ddRPA) platform. In this system, the number of copies with ddRPA was measurable as it was concordant to the number of copies measured with digital droplet PCR (ddPCR) [95]. While Tortajada-Genaro *et al.* presented a platform which performed isothermal RPA on digital versatile discs (DVDs) (Figure 8B). In this work, the presence of amplification products modified the light intensity of the scanning laser of the DVD drive, which made the amplification products detection possible [96].

#### 4.1.4. DNA Hybridization

DNA hybridization has been an important tool for genetic analysis and diagnostics. Centrifugal microfluidics has been used in dynamic DNA microarray hybridizations to decrease reagent consumption and facilitate target diffusion to the substrate surface.

Peytavi *et al.* presented a centrifugal microfluidics-based DNA hybridization system that increased the hybridization signal about tenfold compared with a passive system. In this system, capillary valves were used to achieve the sequential release of sample, wash buffer, and rinse buffer by increasing the rotation speed. The authors successfully discriminated four clinically relevant *Staphylococcus* species that differed by a single-nucleotide polymorphism in only 15 min [97]. Jia *et al.* similarly presented a hybridization microfluidic system in which self-assembled DNA oligonucleotide monolayers on gold pads patterned on glass slides were used for capture probes and enzymatic-labeled fluorescence was used for detection. Capillary valves were also used to achieve the sequential reagent loading. The fluorescence intensity increased up to threefold compared with the passive hybridization assays [98].

Peng *et al.* presented a DNA hybridization system based on numerous radial and spiral channels. The radial channels were used for the first step of DNA probe immobilization, and the spiral channels were used for the second step of DNA hybridization [99]. These increased channel dimensions led to a high hybridization rate.

Roy *et al.* presented a highly integrated platform in which thermoplastic elastomer (TPE) was used for disc fabrication instead of PDMS. Because of the low-temperature and pressure-free assembly and bonding properties, regents storage and loading can be operated more easily on this kind of cartridges. Here, a complete assay including cellular lysis, PCR, amplicon digestion, and microarray hybridization was demonstrated on a single disc, thus proving that integrated biomedical assays can be operated well on this kind of chips [100]. TPE is now recognized as an alternative material for centrifugal microfluidics.

**Figure 9:**
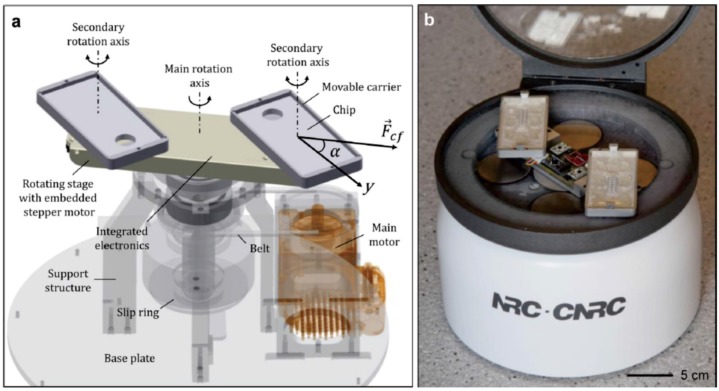
Articulated centrifugal platform. Figure reprinted with permission from [101].

Geissler *et al.* presented a novel articulated centrifugal platform for a colorimetric DNA detection based on cloth-based hybridization array system (CHAS) (Figure 9). In this articulated LOAD platforms, the orientation of the chip with respect to the centrifugal force field could be changed. Based on this platform, new siphon valving and fluid relocation were also demonstrated [101].

### 4.2. Blood Analysis 

Parameters such as glucose, electrolytes, proteins, and lipids in blood can provide much information about the condition of the human body. Integrating the measurement of such parameters into a centrifugal microfluidics cartridge makes point-of-care possible and hasten blood-based diagnoses. In addition, the decreased turnaround time for laboratory tests offers the opportunity to better monitor a patient’s health and decrease unnecessary treatments and hospital costs [102]. We divide blood analysis based on centrifugal microfluidics into two parts: blood plasma separation and blood-based clinical applications.

#### 4.2.1. Blood Plasma Separation

Human blood, which is composed of leukocytes or white blood cells, erythrocytes or red blood cells, and platelets and plasma, contains critical information about how a body is functioning [103,104]. As blood cells may provide a great level of noise that falsifies the results of a biochemical test, most blood tests are performed using plasma or blood serum [105]. Therefore, the extraction of plasma from whole blood is the first preparative step in many assay protocols and of major importance in medical diagnostics. Traditional separation methods such as centrifugation or sedimentation are typically time consuming and require large blood volumes (mL) [106]. These separation processes also fail to allow seamless integration with subsequent assay steps to avoid expensive interconnection techniques and manual intervention.

The methods for microfluidics-based blood plasma separation can be classified into three groups: centrifugation [71,107,108]; inertia force [109], such as the Zweifach-Fung effect [17,110,111] or pinch flow effect [112]; and filtration involving microparticles [113], membranes [114], micropillars [115], and narrow channels [116]. Specifically, for the two passive separation methods based on inertial force, Zweifach-Fung effect stipulates that as the red blood cells flow through a bifurcation region they tend to travel into the faster flow rates channels. On the other hand, pinch flow effect ensures that as blood and another liquid flow are continuously introduced into a microchannel having a pinched segment blood cells will be separated perpendicularly to the flow direction according to their sizes. However, all of these methods have drawbacks. For the first method, the efficiency of the decantation of purified plasma is often not very high. For the second method, the microfluidic channel usually must be long and narrow and have a high flow rate. For the filtration method, clogging of the blood cells caused by filtration units often leads to a high percentage of damaged cells. To make the plasma separation techniques more suitable for point-of-care applications, the system should not require any sophisticated or cumbersome equipment and maybe passive separation techniques are a better choice. However, generally passive systems may not provide high separation efficiency.

Kuo presented a plasma separation and preparation centrifugal microfluidic platform. Here, plasma was first separated from the whole blood based on inertial force. The separated plasma was then aliquoted into two branches and a creatinine test was demonstrated in the detection chamber [117].

#### 4.2.2. Clinical Applications Based on Blood

Nwankire *et al.* presented a liver function screening system. Blood plasma was separated from finger-prick samples. Centrifugo-pneumatic valving was then used for plasma metering and aliquoting the plasma into separate reaction chambers. The reactions were quantified via colorimetric measurements, and the entire liver assay panel was completed in less than 20 min [118].

Riegger *et al.* presented a hematocrit level determination system that involved three steps: priming, metering, and sedimentation. The hematocrit was indicated at the sharp phase boundary between the plasma and segregated cellular pellet on a disc-imprinted calibrated scale. Hematocrit determination of human blood was conducted within 5 min at a high degree of linearity and accuracy with the use of this system [119]. Lin *et al.* presented a prothrombin time (PT) test system. In this system, plasma was extracted from whole blood samples and a rapid mixing of plasma and PT reagent was conducted within 1 s. The test results were gathered within 2 min [120]. Later, the same group reported another PT test platform in which alternating clockwise and counter-clockwise rotation was used to extract plasma form whole blood [121]. Kuo *et al.* presented a LOAD platform for plasma separation and subsequent plasma mixing with suitable regents. Rectangular corner feature mix channels were used to improve the mixing efficiency. The researchers conducted PT test with this platform [122].

**Figure 10:**
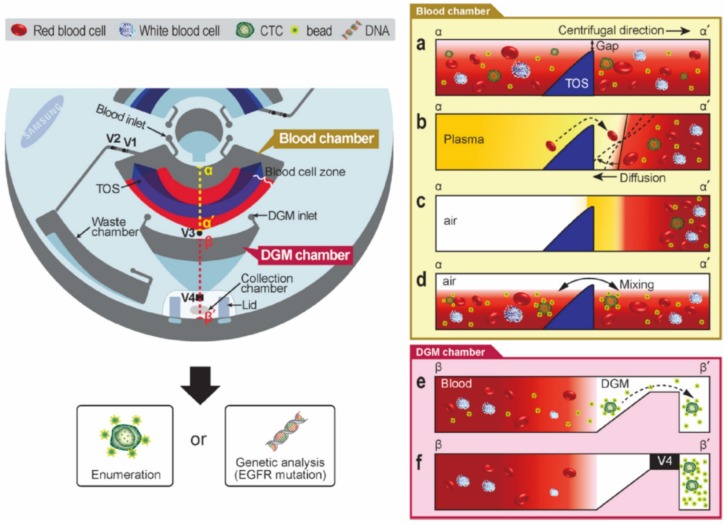
Triangular obstacle structure disc for CTC isolation. Figure reprinted with permission from [123].

Circulating tumor cells (CTCs) have been associated with clinical outcomes in various malignancies, and the isolation of CTCs from whole blood has attracted a great deal of research attention. Park *et al.* presented a CTC isolation system that used a density gradient medium (DGM) (Figure 10). Here, CTCs were bound to the microbeads covered with anti-EpCAM to discriminate the density of the CTCs and blood cells and thus were settled only under the DGM layer. Active laser-actuated ferrowax microvalves were used for fluidic routing [123]. Lee *et al.* presented a size-selective system that isolated CTCs from whole blood samples using a thin membrane with a pore size of 8 mm [124]. Nwankire developed a system for cancer cell quantification. In this paper, plasma was first separated from the whole blood and cancer cells were extracted from the plasma subsequently. The captured cancer cells were detected with the use of an electrochemical method as cell capture resulted in a shift in the impedance between two electrodes [125].

As HIV virus primarily infects the CD4+ T lymphocytes, isolation and counting CD4+ cells is the most important test used in HIV diagnosis and treatment. Ramachandraiah *et al.* presented a single cell resolution imaging LOAD system in which CD4+ cells isolated from whole blood were counted for rapid and low-cost HIV diagnostics. Here, the CD4+ cells were first captured by discs modified with antibodies. With the help of improved DVD-based laser scanning microscope (DVD-LSM), based on the increased light scattering of captured biomolecules, the imaging with a resolution down to 1mm was demonstrated successfully [126]. Glynn *et al.* also demonstrated a centrifugo-magnetophoresis platform for CD4+ cells insolation from the whole blood. In this system, CD4+ cells bound with paramagnetic microparticles were deflected into a designated reservoir by the lateral magnetic field while unbound cells followed the radial vector. The reported separation efficiency was up to 92% [127].

### 4.3. Immunoassays

Based on the highly specific affinity of antibodies to antigens, immunoassays have become important tools in clinical diagnostics, biological and biochemical disease studies, drug development, and environmental analyses [1]. For the most commonly used immunoassays format, the antibody or antigen is bound to the solid phase. When the fluid containing the analyte flows through the surface of the solid phase, the analyte is captured by the antibody or antigen. After this, the secondary antibody or antigen labeled with a tracer is often used to bind the captured analyte to build up a sandwich-type structure. The binding tracer involved in fluorescent or enzymatic labels, colloidal gold, radioisotopes, or magnetic labels is quantified to quantify the captured analyte.

Although immunoassays have been successfully performed in laboratory, the tests can be labor intensive and consist of a large number of manual processing steps. “Long incubation times are a bottleneck for the process, and the tedious and lengthy protocols often result in errors and inconsistent results.” [7] As platform-based automation helps to decrease costs and ensure consistent results, the integration of immunoassays into centrifugal microfluidic platforms is very attractive. Simultaneous parallel immunoassay analyses are also performed easily on centrifugal microfluidic platforms.

#### 4.3.1. Enzyme-Linked Immunosorbent Assay (ELISA)

As a very important format for immunoassays, the enzyme-linked immunosorbent assay (ELISA) uses an enzyme as a tracer. In an ELISA, the enzymatic reaction product yielded from a substrate solution within a designated time is often measured to quantify the amount of the enzyme.

Lai *et al.* presented a centrifugal microfluidic cartridge for ELISA-based immunoassays where the sequential loading of sample, washing, conjugate, and substrate solutions was carried out via a capillary valving system. The detection was achieved using an inverted fluorescence microscope. Analysis of rat immunoglobulin G from a hybridoma culture showed that the microchip-based ELISA had the same detection range as the conventional method on the 96-well microtiter plate with less reagent consumption and shorter assay time over the conventional method [33].

Lee *et al.* presented a fully automated ELISA system to test infectious diseases from whole blood. In this system, laser irradiated ferrowax microvalves were used for sequential loading and shake-mode mixing was implemented to mix beads with the plasma, detection probe, and washing buffers. The concentrations of the antigen and antibody of Hepatitis B virus, HBsAg, and Anti-HBs were measured in parallel on a single cartridge [128]. Lee *et al.* presented another fully integrated device to perform both multiple biochemical analysis and sandwich-type immunoassay simultaneously on a disc. In this device, a lipid test panel composed of six different kinds of biochemical analyte was demonstrated to detect CK-MB (muscle and brain fraction of creatine kinase) as a biomarker for recent heart attacks [129]. 

Park *et al.* presented a centrifugal microfluidic layout to simultaneously detect high sensitivity C-reactive protein, cardiac troponin I, and N-terminal-pro-B-type natriuretic peptide based on a bead-based sandwich-type ELISA. Active laser-actuated microvalves were used to isolate the fluidic channels after the reaction chambers were flushed with common liquids simultaneously. Compared with the conventional ELISA, this assay had a similar limit of detection and dynamic range but required a smaller sample volume and shorter process time [130]. Kim *et al.* presented a bead-based ELISA using the electrochemical detection method. In this ELISA, the horseradish peroxidase enzyme was conjugated to a secondary antibody and catalyzed the reaction of tetramethylbenzidine from its reduced form to its oxidized form, after which amperometry was used for detection. The limit of detection of this device for the C-reactive protein proved to be a 17 times improvement over quantification by optical density [131].

Lee *et al.* developed an electrospun TiO_2_ nanofiber LOAD system for the detection of serum proteins. Here, the high specific surface area enabled TiO_2_ nanofiber to capture more antibodies and then improved the sensitivity of the detection [132].

**Figure 11:**
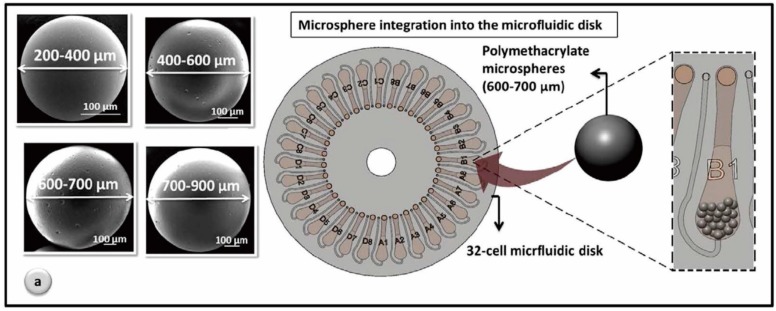
Microfluidic design of microsphere based DV detection ELISA platform. Figure reprinted with permission from [133].

Hosseini *et al.* reported a microsphere based centrifugal microfluidic ELISA platform for the detection of dengue virus (DV) (Figure 11). Here, microspheres with large specific surface area were used to facilitate biorecognition and microballoon mixing was used to ensure more target analyte to be bound on the surface [133].

#### 4.3.2 Other Immunoassay Formats

In addition to the ELISA, fluorescent immunoassays that apply colored beads as the solid phase have also been introduced in centrifugal microfluidics. Riegger *et al.* presented color-multiplexed fluorescent immunoassays. First, a monolayer of color-encoded beads identified by incorporated color tags (dyes or luminescing quantum dots) was aggregated into a detection chamber. The reaction-specific fluorescence signal was then quantified by an optical read-out device. Hepatitis A and tetanus assays were successfully demonstrated in this system [134]. Burger *et al.* presented a combination of color-coded multiplexing with beads, captured in V-shaped cups [135]. Czilwik *et al.* presented a human C-reactive protein (CRP) magnetic chemiluminescent immunoassay (MCIA) centrifugal microfluidic platform [136]. Here, the same gas-phase transition magnetophoresis method, which was first reported in [73], was used to transport magnetic capture microparticles between adjacent chambers.

Hemmi *et al.* demonstrated label-free immunoassays on a centrifugal cartridge. In this system, a CD-type surface plasmon resonance sensor was successfully used in an immunoassay of immunoglobulin A [137].

### 4.4. Other Biomedical Applications

There are also many cell-based assays in the centrifugal microfluidic platforms. These cell-based assays often involve cell isolation or capture and subsequent analysis of the isolated cells [138,139,140]. Cell isolation enables researchers to study and analyze single cells in a defined environment and holds much promise as a research tool, especially in the pharmaceutical field [1,7]. Specifically, Espulgar *et al.* developed a LOAD platform for single-cell level cardiomyocyte-based drug profiling and screening. Here, isolated single and groups of neonatal rat cardiomyocytes was trapped by centrifugation in one chip. After stopping the centrifugation, the cells were observed under a microscope and movies of the beat motion were also recorded [141]. The same group also presented a similar LOAD system to monitor cell–cell interaction of rat cardiomyocytes [142].

Ren *et al.* presented a simulation of an emulsification and separation LOAD platform which enabled different kind of cells to be encapsulated and incubated by droplets [143]. This work has given a theoretical reference for the design of this kind of platforms. Ouyang *et al.* presented a multilayer polyester film centrifugal platforms in which protein level in human blood plasma were quantified. This platform also made parallel aliquoting, reciprocating mixing and larger scale integration more easily in centrifugal microfluidics [144]. Wang *et al.* presented a LOAD platform for screening protein crystallization conditions. In this device, vapor diffusion often used in conventional protein crystallization was realized with the help of capillary valves and vapor-diffusion champers [145]. Schuler *et al.* implemented a high internal volume fractions droplet generation platform [146]. Biomedical and chemical applications such as drug delivery, clinical diagnostics and material synthesis may benefit from this method.

Aeinehvand *et al.* presented a system which was used to detect the DV with the help of microballoon mixers. It proved that the effect of microballoon mixing enhanced the sensitivity of DV detection [147]. Additionally, Antunes *et al.* reported a centrifugal microfluidic platform to quantify the NS1 dengue biomarker in serum. Here, coated magnetic nanoparticles were used to bind target antigen NS1 and then form nanoclusters. Later, the amount and size of the nanoclusters was measured to quantify the target concentration [148]. While, Koh *et al.* developed a sensitive botulinum toxin detection system. In this system, toxins were first bound to antibody-laden capture particles. These particles were then sedimented through a density-media and were quantified by laser-induced fluorescence [149]. Schroder *et al.* presented a LOAD platform for rapid and sensitive detection of bacteria from urine [150], while Kim *et al.* used their LOAD system for on-site quantification of microalgal lipids [151].

## 5. Conclusions and Outlook

This paper gives an overview of the development of centrifugal microfluidics technologies based on advanced unit operations and their biomedical applications. The advanced unit operations include mixing, valving, flow switching, metering, and sequential loading. Their biomedical applications include nucleic acid analysis, blood analysis, immunoassays, and other biomedical applications. This introduction should give readers a clear picture of the current development of centrifugal microfluidics.

**Figure 12:**
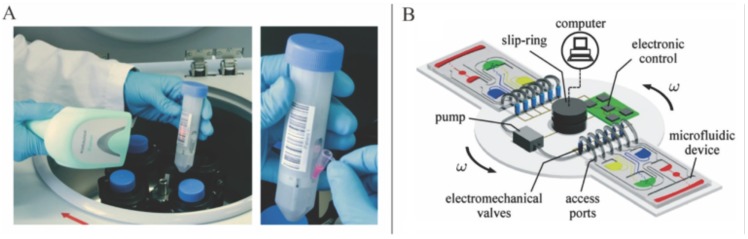
(**A**) LabTube system for centrifugal microfluidics. Figure reprinted with permission from [152]. (**B**) Active pneumatic control of centrifugal microfluidic flows. Figure reprinted with permission from [156].

Although centrifugal microfluidics has developed rapidly over the past decades, it faces many challenges. In terms of point-of-care diagnostics, as the rotation system is cumbersome compared with the disc-shape chip, centrifugal microfluidic devices have to sacrifice its portability to some extent. Considering the widely used fluorescence detection methods for which bulky optical devices are required, the situation may become worse in centrifugal microfluidics. Many efforts have been made to solve this problem. For example, LabTube system with the help of laboratory centrifuges (Figure 12A) [152,153] and lab on DVDs system using DVD discs and commercial drives [96,126] have been suggested to make centrifugal microfluidic devices more universal and portable. In NA analysis, simple and compact colorimetric detection is used instead of fluorescence detection in some cases as a result of the development of isothermal amplification methods [89,91]. When it comes to cost-related issues, considering that the rotation system is reusable, the actual running cost for buying molded plastic discs should not be high. However, the cost of manufacturing the disposable chip, especially for the parts that contains temperature control modules often used in NA analysis, has to be considered. Furthermore, the techniques of storage of reagents should also be improved to ensure that the devices can function well in some extreme environmental conditions. According to the review of reagent pre-storage in microfluidics [154], high precision, low-cost manufacturing, and longtime storage of reagents are still issues not fully resolved. Large-scale integration of the disc-shaped chip is also difficult due to its distance away from the rotation center, flow direction and radius limitations. Although one may argue that this issue can be solved by pumping liquids from a radially outward direction to a radially inward direction [29,50,83,155], the number of the workflows requiring different pre-stored reagents along the radial direction is still limited due to the finite length of the radius. This problem may be solved through the introduction of multi-layer LOAD.

In the future, the development of centrifugal microfluidics may focus on improving the unit operations to widen their applications and promote their integration ability. One of way to improve unit operations is to integrate multi-manipulation strategies into one platform. Clime *et al.* presented a good example in which a regulated pressure pump and a programmable electromechanical valving system were integrated into the centrifugal microfluidic platform (Figure 12B) [156]. Burger *et al.* also presented a good example for integrating the optical manipulation strategy into the LOAD system [138]. However, as mentioned earlier, the use of optical devices may sacrifice the advantage of compactness and portability. More microelectronic devices can also be integrated into the centrifugal microfluidic platform in order to increase its versatility. However, the cost of integrating microelectronic devices has to be considered with care.
